# Learner perceptions of AI-mediated affective support in L2 English learning: A cross-sectional survey

**DOI:** 10.1371/journal.pone.0360925

**Published:** 2026-10-27

**Authors:** Qiuxi Zhuo

**Affiliations:** School of Foreign Studies, Fuzhou University, Fuzhou, China; Central South University, CHINA

## Abstract

Emotional barriers can shape participation in second-language (L2) learning, yet evidence about affective support attributed to artificial intelligence (AI) remains conceptually and methodologically uneven. This cross-sectional online survey examined university-affiliated L2 English learners’ reported emotional challenges, perceptions of AI-mediated affective support, and views of the boundary between AI and teacher support. The Chinese-language, researcher-developed questionnaire used categorical and multiple-response items rather than psychometric scales. Of 425 submissions, one non-university respondent and four respondents younger than 18 years were excluded, yielding an adult analytical sample of 420. The non-probability convenience sample was institutionally concentrated and should not be interpreted as representative of all Chinese university L2 learners. Frequencies, percentages, and three Pearson chi-square tests were calculated. Helplessness (59.05%), fatigue (55.95%), and anxiety (53.33%) were the most frequently reported emotional challenges. Overall, 67.38% perceived that AI could provide affective support, and 84.76% reported using AI for learning challenges. Immediate feedback was the most frequently selected support mechanism (79.52%), while relief of learning pressure or anxiety was the most frequently selected function (70.71%). Perceived AI affective support was associated with reported motivation change, χ²(2) = 53.27, p < .001, Cramér’s V = .356, and interest change, χ²(2) = 59.57, p < .001, V = .377. The combined belief that AI might fill or replace teacher affective support was associated with continuing need for teacher support, χ²(1) = 72.97, p < .001, φ = .417. These results indicate patterned learner perceptions, not causal effects or evidence that AI possesses genuine emotion. AI may offer scalable, low-pressure support for routine learning, whereas teachers remain important for relational, contextual, and pedagogical judgment. Longitudinal studies using validated measures are needed.

## Introduction

Second-language (L2) learning is simultaneously cognitive, social, and affective. Positive emotions can broaden attention and sustain engagement, whereas anxiety and other negative emotions can narrow attention, inhibit participation, and complicate language processing [1,2]. Krashen’s affective-filter account made this relationship prominent in second-language acquisition by proposing that anxiety, low motivation, and low self-confidence can impede the use of otherwise comprehensible input [3]. Although the affective filter is best treated as an interpretive lens rather than a directly observed mechanism, it focuses attention on an important pedagogical problem: learners may have access to linguistic input yet remain emotionally unable or unwilling to engage with it.

Affective scaffolding provides a complementary account of how support in the learning environment can help learners manage this problem. Building on sociocultural views of assisted development [4], research on caring relationships and classroom interaction has described scaffolding as involving not only cognitive assistance but also support for learners’ emotions, motivation, and sense of safety [5–7]. Such support has traditionally been associated with teachers and peers. However, affective scaffolds can also be embedded in material and technological arrangements that shape how a person regulates emotion [8]. This broader view makes it possible to ask whether learners experience some AI functions—such as timely feedback, adjustable pacing, repeated practice, or encouragement—as affectively supportive.

The distinction between affective support and genuine emotion is essential. An AI system need not feel empathy for a learner to be perceived as reducing evaluative pressure or making help available at the moment of difficulty. Conversely, simulated encouragement does not establish that an AI system understands a learner’s personal history, classroom relationships, or cultural context. Research on affective computing has long examined how technologies detect or respond to affective cues [9,10], but the present study concerns learner-attributed support rather than machine consciousness, emotion recognition accuracy, or therapeutic efficacy.

This distinction matters because recent AI-assisted L2 research often reports motivation, enjoyment, engagement, confidence, anxiety, or stress, but studies differ substantially in design, instruments, tools, and learning contexts [11]. Intervention studies can estimate change under specified instructional conditions, whereas cross-sectional perception surveys describe how learners interpret their experiences. Conflating these forms of evidence risks converting perceived usefulness into causal claims. The present study therefore adopts a deliberately bounded aim: to describe adult university-affiliated L2 English learners’ reported emotional challenges, the mechanisms and functions they attribute to AI, and their views of the boundary between AI-mediated and teacher-provided support.

Two distinctions sharpen this problem. First, an AI affordance is not itself an affective outcome: immediate feedback or private access may be available, but its emotional significance depends on the learner, task, response quality, and surrounding instructional context. Second, affective support is not interchangeable with convenience, satisfaction, or frequent use. A tool may be easy to access without helping a learner regulate pressure or re-engage with a difficult task. These distinctions require a survey to map each inference to an observable response rather than treat several favorable judgments as one latent construct. They also motivate the present study’s emphasis on learner attributions, explicit denominators, and a separate analysis of the AI–teacher boundary.

## Theoretical framework

### Affective filter as an interpretive lens

The affective-filter hypothesis proposes that motivational and emotional conditions influence whether available input is effectively engaged with [3]. In the present study, the hypothesis is not operationalized as an internal filter score. Instead, it provides a cautious interpretive lens for examining reported anxiety, helplessness, fatigue, motivation, and interest. A learner’s selection of anxiety or enhanced motivation is therefore treated as a report about experience, not as direct measurement of a neurological filter or proof of language acquisition.

This bounded use is important because the questionnaire did not employ a validated anxiety, motivation, or confidence scale. It identified the relative frequency of selected emotional challenges and reported directions of change. The theoretical question is consequently whether the pattern of selections is consistent with the proposition that perceived access, feedback, and reduced evaluation pressure can support engagement—not whether AI caused a measurable change in a latent affective construct.

### Affective scaffolding as a theoretical lens and learner-perceived affective support as the empirical construct

Vygotskian scaffolding emphasizes contingent assistance within a learner’s developing capacity [4]. Later accounts foregrounded the relational and emotional conditions through which assistance becomes usable [5–7]. Rosiek described emotional scaffolding as pedagogical activity at the intersection of student emotion and subject matter [6], while Meyer and Turner emphasized teacher-initiated interactions that support productive emotional experience during learning [7]. In L2 settings, teacher affective scaffolding has been associated with engagement and psychological well-being [12].

Colombetti and Krueger’s account broadens affective scaffolding beyond direct interpersonal care to include material arrangements that help organize or regulate affect [8]. This does not make technology equivalent to a teacher. It instead permits an analytic distinction between the source of support and its perceived function. AI may provide a repeatable interface, immediate response, or private practice space; a teacher may interpret context, build trust, recognize sustained difficulty, and exercise pedagogical and ethical judgment.

The word scaffolding also imposes conceptual limits. In educational accounts, support is related to an identifiable learning demand, adjusted to the learner, and oriented toward participation that may later become more independent [4–7]. AI interaction can approximate some of these conditions through repeated prompting, adjustable pacing, and responsive feedback, but this survey did not observe diagnostic adjustment, fading, transfer, or subsequent performance. The term is therefore used in a bounded, perception-based sense: participants identified features and functions that they experienced or imagined as supporting the emotional conditions of task engagement. Whether those supports were pedagogically well calibrated remains an empirical question beyond the present design.

For this study, learner-perceived AI-mediated affective support is operationalized as task-contingent support attributed to AI tools that may help manage the emotional demands of L2 learning through availability, immediate feedback, personalized planning, progress visibility, encouragement or reward, and low-evaluation practice. This construct is interpreted through affective scaffolding theory, but the survey does not directly observe pedagogical scaffolding, diagnostic adjustment, fading, transfer, or learning outcomes. Five linked components were examined: perceived availability of affective support (item 8), perceived mechanisms (item 11), perceived functions (item 12), reported directions of motivation and interest change (items 13–14), and the perceived boundary between AI and teacher support (items 18–19). These components are categorical indicators in an operational model; they are not reflective items forming a psychometric scale.

The resulting model moves from technological affordances to learner-attributed functions and then to reported affective directions, with teacher support treated as a complementary boundary condition rather than an obsolete alternative (Fig 1).

**Fig 1:**
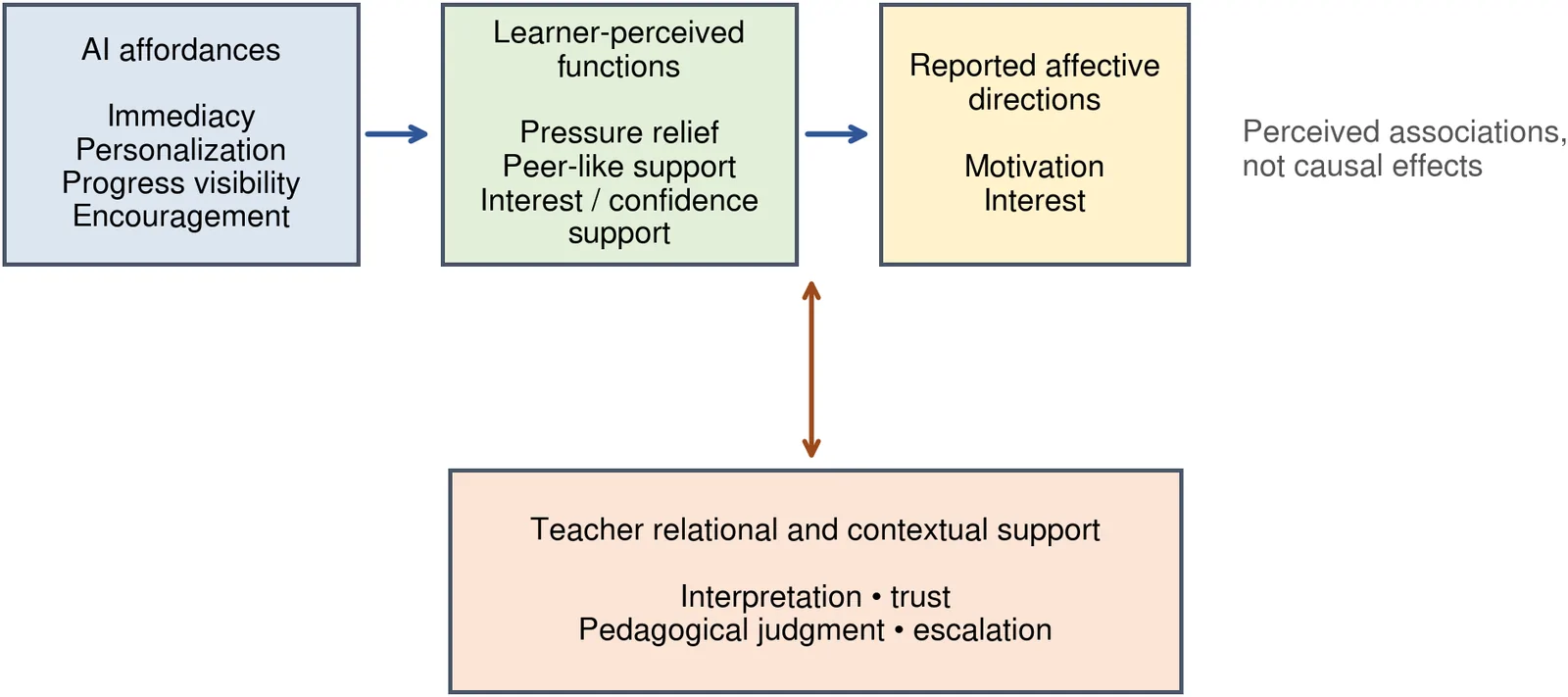
Conceptual and operational model of learner-perceived AI-mediated affective support interpreted through affective scaffolding theory.

Technological affordances are interpreted through learner perceptions, which may be associated with reported affective directions. Teacher relational and contextual support is modeled as a complementary boundary rather than a replaceable equivalent.

### Empirical studies on AI and L2 learner emotions

A recent systematic review of 37 empirical studies found that anxiety and enjoyment dominated research on emotions in AI-assisted English learning, while interest and other affective variables received less attention [11]. The review also showed that higher education was the dominant setting and that positive and negative experiences could coexist. Qualitative work similarly documents mixed emotional responses: learners have reported motivation, excitement, engagement, and confidence alongside frustration, anxiety, and stress [13,14]. This pattern cautions against treating AI as uniformly emotion-enhancing.

Intervention studies provide greater leverage for assessing change under particular conditions. AI-driven chatbots have been associated with improved behavioral, cognitive, and emotional engagement in a controlled intervention with Chinese EFL learners [15]. A 16-week Gemini-supported writing intervention reported improved writing proficiency and reduced writing anxiety relative to conventional instruction [16]. Other longitudinal or experimental work has reported growth in positive achievement emotions and flow [17], and increases in positive emotions with reductions in negative emotions following multimodal AI-supported instruction [18]. AI-supported English speaking has also been linked to enjoyment and willingness to communicate [19]. These studies show what may occur in structured instructional programs, but their findings cannot be assumed to generalize to unstructured use of diverse AI tools.

Perception-focused studies address a different question. Learners have described generative AI as influencing emotional engagement and goal orientation [20], while longitudinal research on translanguaging in GenAI-enhanced classes connects AI-mediated practice with trajectories of achievement emotions and academic engagement [21]. Together, these studies indicate that immediacy, adaptive interaction, multimodality, and language flexibility may be affectively salient. However, many studies examine a single tool, skill, or planned course, leaving less evidence about how heterogeneous university learners understand support across translation software, language apps, speech tools, and domestic or international chatbots.

The evidence base also differs in how emotion is operationalized. Validated scales, retrospective questionnaires, interviews, classroom observations, and platform traces do not answer the same question, and positive change observed in a designed intervention may reflect the combined influence of the AI tool, teacher orchestration, additional practice, novelty, and course structure [11,15,16-18,21]. Perception surveys have less leverage for causal inference, but they can reveal which mechanisms learners recognize across everyday tool ecologies and which sources of support they regard as complementary. This descriptive contribution is especially relevant when participants use multiple categories of AI rather than a single standardized system. The trade-off is reduced technical specificity: a cross-tool pattern cannot identify which model, interface, or feedback design produced a particular response.

Teacher support remains a necessary comparison. Teacher affective scaffolding is relational and pedagogically situated [12], and teacher communication behaviors can shape students’ willingness to attend AI-enhanced classrooms [22]. The empirical question is therefore not simply whether AI or teachers are preferred. It is where learners locate the boundary: routine immediacy and private practice may be delegated to AI, whereas relationship-building, interpretation of complex needs, and accountable judgment may remain human responsibilities. Table 1 summarizes representative empirical studies across different research designs, highlighting how intervention, longitudinal, perception-focused, and teacher-support studies address different aspects of AI-related emotional experiences in L2 learning.

**Table 1:** Summary of empirical evidence informing the present study.

Evidence type	Example studies	Design / focus	Relevance to the present
Systematic review	Zhang & Liu [11]	Review of empirical studies on emotions in AI-assisted English learning	Shows that anxiety and enjoyment dominate existing research and that positive and negative emotions coexist
Qualitative/perception studies	Yang & Zhao [13]; Xin & Derakhshan [14]	Learners’ perceived emotions and emotion-regulation experiences in AI-supported L2 contexts	Shows mixed emotional responses, including motivation, confidence, anxiety, and frustration
Intervention studies	Wang & Xue [15]; Derakhshan [16]	AI chatbot or GenAI-supported instructional interventions	Provides stronger evidence of change under designed instructional conditions
Longitudinal / developmental studies	Qin & Derakhshan [17]; Derakhshan & Park [18]	Achievement emotions, flow, and positive/negative emotions in AI-enhanced classrooms	Shows that affective change can be studied longitudinally with stronger designs
AI speaking and communication	Huang & Zou [19]	AI-supported English speaking, enjoyment, willingness to communicate	Indicates that AI interaction may be affectively salient in oral practice
Teacher support in AI-enhanced contexts	Derakhshan & Lalli [22]	Teacher communication and students’ willingness to attend AI-enhanced classrooms	Supports the need to examine AI–teacher complementarity rather than replacement

Note. Academic field / major was collected as free-text information but is not reported in detail because categories were highly heterogeneous and, in combination with institution and educational stage, could increase re-identification risk.

Three gaps follow from this comparison. First, AI-mediated affective support is often discussed without a transparent operational link among technological affordances, learner-attributed affective functions, reported affective directions, and teacher-support boundaries. Second, causal evidence from interventions and descriptive evidence from perception surveys are not always clearly distinguished. Third, the AI–teacher boundary is frequently discussed normatively rather than examined through linked learner responses. The present cross-sectional survey addresses these gaps descriptively and associationally, not by claiming intervention effects, but by mapping which response categories support each inference.

### The current study

Guided by the operational framework and the gaps above, the study addressed the following research questions:

RQ1. What negative emotions do adult university-affiliated L2 English learners report, and in which learning contexts do they occur?
RQ2. What mechanisms and functions do learners attribute to AI-mediated affective support, and how is perceived AI affective support associated with reported motivation and interest change?
RQ3. How do learners perceive the boundary between AI-mediated and teacher-provided affective support?

RQ1 was addressed using Q5 and Q6 through descriptive frequencies and percentages. RQ2 was addressed using Q8 and Q11–Q14 through descriptive summaries and two Pearson chi-square tests linking perceived AI affective support with reported motivation and interest change. RQ3 was addressed using Q18–Q19 through descriptive summaries and a Pearson chi-square test linking the combined fill-or-replace item with continuing need for teacher support.

## Materials and methods

### Study design

This study used a cross-sectional online survey containing closed-ended categorical items, multiple-response items, and open-response fields. The formal analysis reported here is quantitative and uses only the closed-ended and multiple-response items. No instructional intervention, experimental manipulation, control group, interview study, or longitudinal follow-up was conducted. Open responses were not subjected to a preregistered or independently coded qualitative analysis and are not used as evidence in this article. Accordingly, results are interpreted as self-reported perceptions and associations rather than causal effects.

### Participants and recruitment

The author used non-probability convenience sampling and circulated a Wenjuanxing (Questionnaire Star) link through WeChat groups. Eligible participants were university-affiliated L2 English learners at undergraduate, master’s, doctoral, or postdoctoral stages. Participants received RMB 1 through WeChat after completing the survey.

The survey received 425 submissions. One submission from a person who was not a university learner was removed during eligibility screening, leaving 424 otherwise eligible records. Four of these respondents were younger than 18 years. Although the minors were reported to have discussed participation with parents, no contemporaneous record of guardian permission existed; those four records were therefore excluded from every analysis and from files prepared for sharing. The final analytical sample comprised 420 adults. All eligible adult submissions were retained, and no a priori power calculation was conducted.

The sample was institutionally concentrated: 276 participants (65.71%) were affiliated with Fuzhou University. The study therefore does not treat the sample as representative of all Chinese university learners. Participant characteristics are reported in Table 2.

**Table 2:** Participant characteristics (N = 420).

Characteristic	Category	n	%
Sex	Female	220	52.38
	Male	200	47.62
Age group	18–25	371	88.33
	26–30	26	6.19
	31–40	13	3.10
	41–50	9	2.14
	51–60	1	0.24
Education stage	Undergraduate	328	78.10
	Master’s	48	11.43
	Doctoral	38	9.05
	Postdoctoral	6	1.43
English credential	Gaokao English >125	41	9.76
	CET-4	224	53.33
	CET-6	46	10.95
	TEM-4	47	11.19
	TEM-8	62	14.76
Institution group	Fuzhou University	276	65.71
	Huaqiao University	37	8.81
	Beijing Foreign Studies University	26	6.19
	Other institutions	81	19.29

Note. Examination credentials are non-equivalent and are reported descriptively only.

### Ethics approval and consent

Before recruitment, the study protocol was reviewed and approved by the Academic Ethics Committee of the School of Foreign Studies, Fuzhou University, through its formally authorized representative. The committee did not issue an approval number. The first survey page described the study purpose, voluntary participation, the right to discontinue before submission, confidentiality, academic use of aggregated results, and researcher contact information. No separate consent checkbox was presented. Adult participants gave electronic implied consent by reading the information and voluntarily proceeding to submit the questionnaire.

### Questionnaire development and operationalization

The researcher-developed questionnaire was administered in Chinese. It contained two unnumbered demographic questions followed by 20 numbered items. Items 1–4 covered institution, educational stage, academic field, and English examination credential; items 5–9 covered emotional challenges, learning contexts, perceived AI support, and AI use; items 10–17 covered AI tools, mechanisms, perceived functions, reported motivation and interest change, frequency, duration, and satisfaction; and items 18–20 covered AI–teacher boundaries and expectations. Table 3 maps the analyzed domains to item numbers, response formats, denominators, and permitted interpretations. The Chinese instrument and an English translation of its analyzed items are provided in S1 File.

**Table 3:** Operationalization of analyzed questionnaire domains.

Domain	Item(s)	Format	Analysis denominator	Permitted interpretation
Emotional challenges	Q5	Multiple response	N = 420	Frequency of selected challenges; no intensity score
Learning contexts	Q6	Multiple response	N = 420	Contexts selected for negative emotion
Perceived AI support	Q8	Yes/No	N = 420	Perceived availability of affective support
AI use	Q9	Yes/No	N = 420	Self-reported use when facing learning challenges
AI tools	Q10	Multiple response	n = 356 users	Tools selected by self-reported users
Support mechanisms	Q11	Multiple response	N = 420	Perceived or hypothetical mechanisms
Perceived functions	Q12	Multiple response	N = 420	Selected functions; not validated trait scores
Motivation/interest	Q13–Q14	3-category direction	N = 420	Reported direction of change; not causal effect
Use patterns	Q15–Q17	Categorical	n = 356 users	Frequency, duration, and satisfaction
AI–teacher boundary	Q18–Q19	Binary	N = 420	Fill-or-replace view and continuing teacher need

Note. Q = numbered questionnaire item. Items 11–14 use N = 420 because the survey platform required responses after item 9; therefore, these selections include hypothetical perceptions among non-users.

Before fielding, a computer science specialist informally reviewed selected items and response-option logic. The first 100 submissions served as a soft launch to check completion flow and option functioning. No wording, item, or option was changed after the soft launch; eligible adult soft-launch records were retained because administration and inclusion criteria were unchanged. Neither the informal review nor the soft launch constituted formal expert content validation or psychometric validation.

The questionnaire was not a multi-item psychometric scale and was not designed to estimate latent anxiety, motivation, confidence, or affective-scaffolding scores. Items were categorical or multiple-response questions and were not reflective indicators of a single construct. Cronbach’s alpha and factor analysis were therefore not calculated. Terms such as “confidence support” in the results refer only to selection of the corresponding closed response option, not to a validated confidence measure.

### Procedure, data management, and denominators

The survey did not request names or student numbers. However, Wenjuanxing automatically recorded technical metadata and source information, including IP addresses or platform-source identifiers. These fields were separated from research variables and removed before analysis. Exact institutions, academic majors, timestamps, and free-text responses were also excluded from the aggregate supporting dataset to reduce re-identification risk.

Closed-ended items were configured as required and therefore contained no missing values among submitted adult records. Although the information page stated that questions could be skipped, item-level skipping was not technically enabled for required fields; respondents could still withdraw by closing the survey before submission. This implementation discrepancy is acknowledged as a limitation.

Multiple-response selections were recoded as separate binary indicators. Items 10 and 15–17 were summarized only among the 356 respondents who reported using AI sometimes or often. The questionnaire instructed non-users to skip the AI-tool item, but the platform required later items. Consequently, items 11–14 on mechanisms, functions, motivation, and interest were summarized for all 420 adults and are interpreted as perceptions, including hypothetical perceptions among non-users. Denominators are stated for every table and figure.

### Statistical analysis

Frequencies and percentages were calculated for categorical and multiple-response items. Three targeted Pearson chi-square tests aligned with RQ2 and RQ3 examined (1) perceived AI affective support by reported motivation change, (2) perceived AI affective support by reported interest change, and (3) belief that AI might fill or replace teacher affective support by continuing need for teacher support. Cramér’s V was reported for 2 × 3 tables and phi (equivalent to Cramér’s V for a 2 × 2 table) for the third association. All expected counts exceeded 5. A two-sided α of  .05 was used; because three tests were conducted, results were also checked against a Bonferroni-adjusted threshold of  .0167. No missing-data imputation was performed.

Calculations were independently reproduced in Python 3.12 using pandas and SciPy and cross-checked using formulas in S1 Data. The analysis did not include regression, machine-learning prediction, psychometric scale scores, or formal qualitative coding.

## Results

### Participant characteristics

The final sample included 420 adults: 220 women (52.38%) and 200 men (47.62%). Most were aged 18–25 years (n = 371, 88.33%) and were undergraduates (n = 328, 78.10%). Examination credentials were non-equivalent categories and are used only to describe the sample; they were not treated as an ordinal English-proficiency measure (Table 2).

### Reported emotional challenges and learning contexts

Helplessness was the most frequently selected emotional challenge (n = 248, 59.05%), followed by fatigue (n = 235, 55.95%), anxiety (n = 224, 53.33%), loneliness (n = 76, 18.10%), and the “Other” option (n = 57, 13.57%). Practice activities were the most frequently selected context (n = 255, 60.71%), followed by after-class assignments (n = 243, 57.86%) and the language classroom (n = 183, 43.57%). Because these were multiple-response items, percentages do not sum to 100% (Table 4 and Fig 2).

**Table 4:** Reported emotions, contexts, perceptions, mechanisms, functions, motivation, and interest (N = 420).

Domain	Response	n	%
Negative emotion	Helplessness	248	59.05
	Fatigue	235	55.95
	Anxiety	224	53.33
	Loneliness	76	18.10
	Other	57	13.57
Context	Practice activities	255	60.71
	After-class assignments	243	57.86
	Language classroom	183	43.57
Perception/use	Perceives AI affective support	283	67.38
	Reports using AI	356	84.76
Support mechanism	Immediate feedback	334	79.52
	Personalized learning plan	203	48.33
	Progress visualization	150	35.71
	Encouragement/reward	104	24.76
	Other	17	4.05
Perceived function	Pressure/anxiety relief	297	70.71
	Simulated peer support	150	35.71
	Confidence support	134	31.90
	Interest support	133	31.67
	Filling gaps in teacher support	126	30.00
	Other	18	4.29
Motivation change	Enhanced	281	66.90
	No effect	108	25.71
	Reduced	31	7.38
Interest change	Enhanced	276	65.71
	No effect	111	26.43
	Reduced	33	7.86

Note. Emotions, contexts, mechanisms, and functions were multiple-response items; percentages within those domains do not sum to 100%.

**Fig 2:**
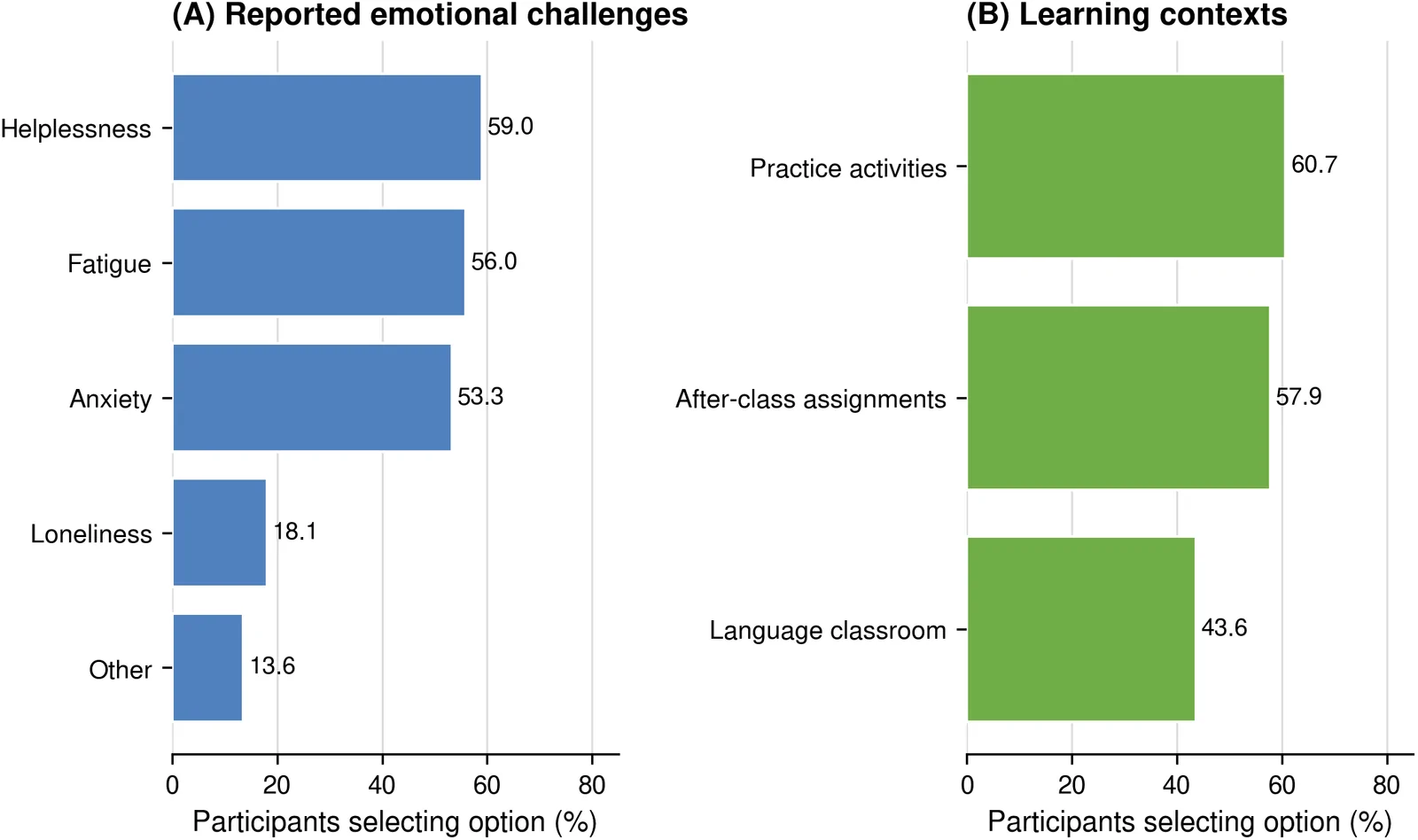
Reported emotional challenges and learning contexts. (A) Learner-selected emotional challenges and (B) learning contexts (N = 420). Items allowed multiple responses; percentages therefore do not sum to 100%.

### AI use and perceived support

A total of 283 participants (67.38%) reported that AI could provide affective support, and 356 (84.76%) reported using AI to address learning challenges. Among the 356 AI users, translation software was most common (n = 286, 80.34%), followed by domestic chatbots (n = 226, 63.48%), foreign chatbots (n = 122, 34.27%), language-learning applications (n = 118, 33.15%), speech-recognition software (n = 84, 23.60%), and other tools (n = 7, 1.97%).

Across the full adult sample, immediate feedback was the most frequently selected support mechanism (n = 334, 79.52%), followed by personalized learning plans (n = 203, 48.33%), progress visualization (n = 150, 35.71%), and encouragement or reward (n = 104, 24.76%). Relief of learning pressure or anxiety was the most frequently selected perceived function (n = 297, 70.71%). Other selections were simulated peer support (n = 150, 35.71%), perceived confidence support (n = 134, 31.90%), interest support (n = 133, 31.67%), and filling gaps in teacher support (n = 126, 30.00%) (Table 4 and Fig 3). These selections describe perceived functions and do not demonstrate objective reduction in anxiety or causal change in confidence. As a user-only sensitivity check, the same descriptive ordering was observed among the 356 self-reported AI users. Immediate feedback remained the most frequently selected perceived mechanism (n = 290, 81.46%), followed by personalized learning plans (n = 178, 50.00%), progress visualization (n = 131, 36.80%), and encouragement or reward (n = 91, 25.56%). Relief of learning pressure or anxiety also remained the most frequently selected perceived function among users (n = 266, 74.72%), followed by simulated peer support (n = 131, 36.80%), confidence support (n = 124, 34.83%), interest support (n = 123, 34.55%), and filling gaps in teacher support (n = 107, 30.06%). This sensitivity check suggests that the full-sample descriptive pattern was not driven solely by hypothetical responses from non-users, although the study still cannot separate experienced and imagined mechanisms for every item.

**Fig 3:**
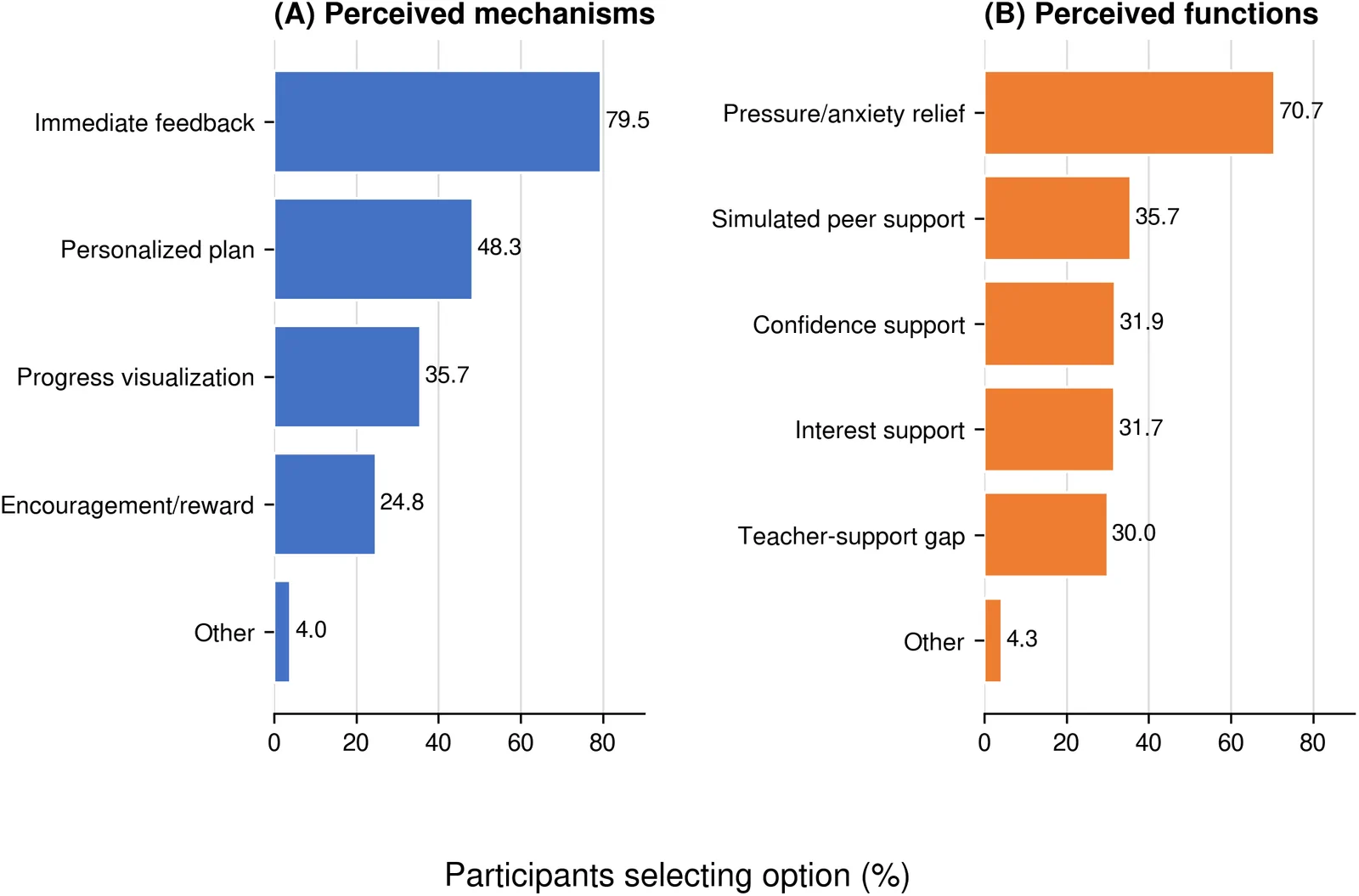
Learner-selected AI support mechanisms and perceived functions. (A) Perceived mechanisms and (B) perceived functions (N = 420). Items allowed multiple responses and include hypothetical judgments from respondents reporting no AI use.

Among AI users, 88 (24.72%) reported use several times daily, 45 (12.64%) once daily, 95 (26.69%) three to five times weekly, 73 (20.51%) one to two times weekly, and 55 (15.45%) rarely. Most sessions lasted less than 30 minutes (n = 246, 69.10%). A total of 256 users (71.91%) were satisfied or very satisfied with their tools (Table 5).

**Table 5:** AI-use patterns among self-reported AI users (n = 356).

Measure	Category	n	%
Frequency	Several times daily	88	24.72
	Once daily	45	12.64
	3–5 times weekly	95	26.69
	1–2 times weekly	73	20.51
	Rarely	55	15.45
Session duration	<30 minutes	246	69.10
	30–60 minutes	87	24.44
	>1 hour	23	6.46
Satisfaction	Very satisfied	62	17.42
	Satisfied	194	54.49
	Neutral	95	26.69
	Dissatisfied	3	0.84
	Very dissatisfied	2	0.56

### Associations with reported motivation and interest

Perceived AI affective support was associated with reported motivation change, χ²(2, N = 420) = 53.27, p < .001, Cramér’s V = .356. Among respondents who perceived AI affective support, 222 of 283 (78.45%) selected enhanced motivation, compared with 59 of 137 (43.07%) among those who did not perceive such support. Perceived support was also associated with reported interest change, χ²(2, N = 420) = 59.57, p < .001, V = .377. Enhanced interest was selected by 221 of 283 respondents (78.09%) who perceived AI support and 55 of 137 (40.15%) who did not. Both associations remained significant at the Bonferroni-adjusted threshold. These cross-sectional associations do not establish that perceived AI support caused the reported changes.

### Perceived AI–teacher boundary

A total of 234 respondents (55.71%) selected that AI might “fill or replace” affective support provided by a language teacher, whereas 186 (44.29%) selected that it could not. Because the item combined “fill” and “replace,” an affirmative response cannot be interpreted as endorsement of complete teacher replacement. Separately, 168 respondents (40.00%) indicated that they still needed or expected additional affective support from teachers.

The fill-or-replace view was associated with continuing need for teacher support, χ²(1, N = 420) = 72.97, p < .001, φ = .417, and remained significant after the Bonferroni check. Among respondents who rejected the proposition that AI could fill or replace teacher support, 117 of 186 (62.90%) still expected additional teacher support. Among respondents who accepted the combined proposition, 51 of 234 (21.79%) still expected additional teacher support (Table 6 and Fig 4).

**Table 6:** Observed contingency counts and targeted Pearson chi-square tests (N = 420).

Association / row group	Reduced / No teacher need	No effect / Teacher need	Enhanced	χ² (df)	Effect
AI support × motivation: No support (n = 137)	15	63	59	53.27 (2)	V = .356
AI support × motivation: Support (n = 283)	16	45	222		
AI support × interest: No support (n = 137)	17	65	55	59.57 (2)	V = .377
AI support × interest: Support (n = 283)	16	46	221		
AI fill/replace × teacher need: No (n = 186)	69	117	—	72.97 (1)	φ = .417
AI fill/replace × teacher need: Yes (n = 234)	183	51	—		

Note. For the 2 × 3 tables, columns are Reduced, No effect, and Enhanced. For the 2 × 2 teacher-boundary table, the first two columns are No teacher need and Teacher need. All p values were < .001. V = Cramér’s V; φ = phi coefficient.

**Fig 4:**
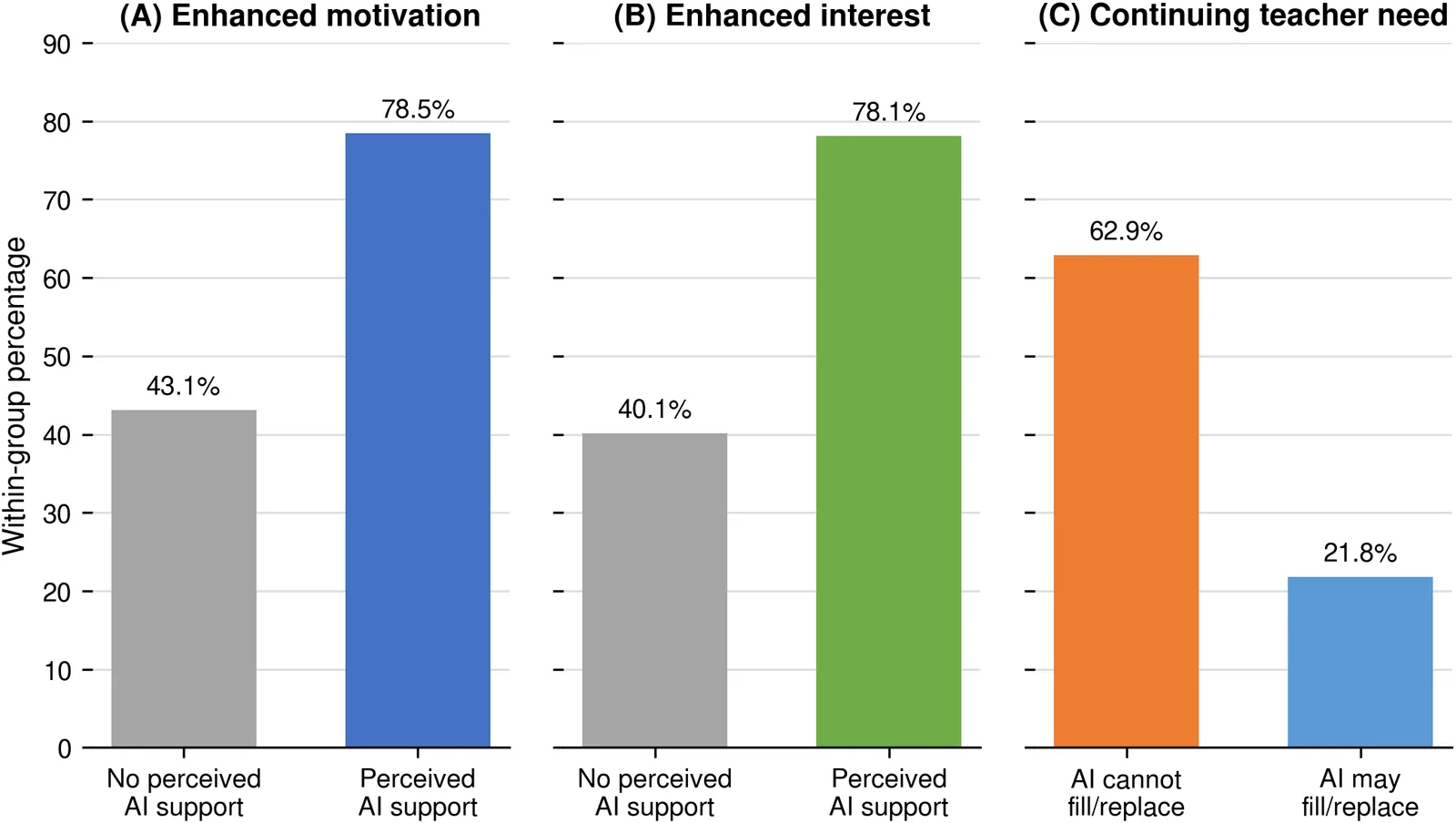
Reported affective directions and continuing teacher need by perceptions of AI support. (A) Enhanced motivation and (B) enhanced interest by perceived AI affective support; (C) continuing teacher need by the combined AI fill-or-replace item (N = 420). Bars show within-group percentages; underlying counts appear in Table 6 and S1 Data.

## Discussion

### Reported emotional challenges through the affective-filter lens

The first research question concerned emotional challenges and their contexts. Helplessness, fatigue, and anxiety were each selected by more than half the sample, and practice activities were the most frequently selected context. Anxiety is prominent in both foundational L2 emotion research [1,2] and recent reviews of AI-assisted English learning [11]. The prominence of helplessness and fatigue in the present data broadens the descriptive picture beyond anxiety alone. It suggests that emotional difficulty may involve perceived lack of control and sustained effort as well as fear of evaluation.

These findings are consistent with the affective-filter proposition that negative emotional conditions can interfere with engagement with available input [3], but the study did not measure input processing, retention, achievement, or a latent filter. The theory therefore helps interpret why learners might value low-pressure assistance; it does not convert the descriptive frequencies into evidence that language acquisition improved.

### AI affordances as learner-perceived affective support

The second research question examined perceived mechanisms and functions. The high use of translation software should also be interpreted cautiously. Q10 identified broad categories of tools, but it did not link each tool category to a specific perceived affective function. Translation software may reduce pressure by supporting comprehension, saving time, reducing uncertainty, or making task completion more manageable; the present survey cannot distinguish among these pathways. The findings therefore identify learner-attributed patterns rather than mechanisms of emotional regulation. Immediate feedback was selected much more often than encouragement or reward, and relief of pressure or anxiety was the dominant perceived function. This pattern locates the affective value of AI less in simulated warmth than in functional availability: a learner can ask, retry, and receive a response without waiting for public evaluation. It also clarifies the operational definition. The perceived support is the learner–tool arrangement that may change how a task feels; this is interpreted through affective scaffolding theory, but it is not evidence that the tool itself experiences emotion or that pedagogically calibrated scaffolding occurred.

The associations with reported motivation and interest were moderate in size. They align with perception-based findings on AI, emotional engagement, and goal orientation [20], and with intervention research reporting gains in engagement [15], reduced writing anxiety [16], positive achievement emotions and flow [17], and increases in positive emotions with reductions in negative emotions [18]. They also accord with research linking AI-supported speaking with enjoyment and willingness to communicate [19]. However, the present design is less causally informative than those interventions. Learners who are already motivated, interested, or favorable toward AI may be more likely to perceive affective support; common-method response tendencies may also contribute.

The effect sizes should be understood as separations between response distributions, not as magnitudes of emotional improvement. Motivation and interest were captured as three directional categories (reduced, no effect, or enhanced) without baselines or validated scale scores, and the same respondents reported both the predictor perception and the outcome direction. Response consistency may therefore inflate the associations. Moreover, the analysis did not adjust for AI-use frequency or other covariates; those factors could partly account for the pattern. These results are best treated as hypothesis-generating evidence that perceived support and reported positive affective directions clustered together in this sample.

The systematic review evidence indicates that AI-assisted learning can evoke both positive and negative emotions [11], and qualitative studies describe frustration, anxiety, and stress alongside motivation and confidence [13,14]. The current study should therefore not be read as demonstrating a universally beneficial emotional effect. The results identify which support options were salient in this sample and which perceptions covaried.

An additional tension concerns emotional comfort and pedagogical value. Private, immediate interaction may make practice feel safer, but lower evaluative pressure does not by itself ensure accurate feedback, productive struggle, or transfer to classroom communication. If learners use AI mainly to avoid human evaluation, short-term relief could coexist with reduced opportunities for interpersonal participation. The practical question is therefore not simply how to maximize comfort; it is how to connect low-stakes AI practice with supported re-entry into communicative learning. This interpretation strengthens the case for teacher oversight while remaining a proposal for future testing rather than a result demonstrated here.

### Revisiting affective support at the AI–teacher boundary

The third research question concerned the boundary between AI and teacher support. The combined wording of item 18 prevents a clean distinction between supplementation and substitution. Nevertheless, the linked item 19 provides an important constraint: even among respondents who selected that AI might fill or replace teacher support, 21.79% still wanted more teacher affective support. The results therefore do not support the claim that learners regard teachers as dispensable.

This pattern is coherent with the distinction between material and interpersonal affective scaffolding [8]. AI can become part of an affective arrangement through predictable access, private rehearsal, and prompt response. Teacher scaffolding, by contrast, is relational, accountable, and situated in an ongoing understanding of learner development [5–7,12]. Research on teacher communication in AI-enhanced classrooms likewise points to the continuing role of human interaction in students’ willingness to attend and participate [22]. The two sources can overlap in perceived function without being interchangeable in mechanism or responsibility.

A bounded collaboration model follows as a practical implication rather than a tested intervention. This model should be treated as a hypothesis for future instructional design and evaluation, not as an empirical model tested by the present survey. AI can support low-stakes repetition, immediate formative feedback, translation, pronunciation rehearsal, and individualized pacing. Teachers can diagnose persistent problems, contextualize AI feedback, establish norms for safe and ethical use, recognize when apparent task difficulty reflects broader distress, and provide relationship-based encouragement. Multilingual and translanguaging designs may further expand access to task meaning and emotional engagement [21], but teacher oversight remains necessary when feedback affects assessment, identity, or well-being.

### Theoretical and practical contribution

The main theoretical contribution is a restricted, reproducible operational mapping of learner-perceived AI-mediated affective support, interpreted through affective scaffolding theory. The model separates affordances (for example, immediacy and personalization), learner-attributed functions (for example, pressure relief or confidence support), reported affective directions (motivation and interest), and the teacher boundary. This prevents the concept from becoming a label for every positive response to technology and makes clear which questionnaire items address each component.

Practically, institutions should avoid presenting AI as a synthetic substitute for teacher care. A more defensible design principle is to use AI for scalable and revisable support while preserving accessible human channels for explanation, relationship-building, and escalation. Because the present sample was dominated by one institution and self-selected users, implementation decisions should be piloted locally and evaluated with validated outcome measures.

### Limitations and future research

First, convenience recruitment through the author’s WeChat groups, the RMB 1 completion payment, and the concentration of participants at Fuzhou University may have produced selection and institutional-context bias. Findings should not be generalized to all Chinese university learners, vocational students, or other national settings.

Second, although items 11–14 were central to the analysis, the survey platform required non-users to answer them after item 9. These results therefore combine experienced perceptions from AI users and hypothetical perceptions from non-users. The significant associations may reflect prior attitudes, reverse direction, shared response method, or unmeasured differences rather than effects of AI. No achievement, behavior, or longitudinal emotional outcome was measured. The added user-only sensitivity check reduces, but does not remove, this interpretive limitation. Future surveys should implement branching logic that routes non-users separately.

Third, the researcher-developed questionnaire used categorical and multiple-response items rather than validated multi-item scales. The informal technical review and 100-response soft launch did not establish content, construct, convergent, or criterion validity. The study cannot estimate the intensity of anxiety, motivation, confidence, or affective scaffolding, and English examination credentials were non-equivalent descriptive categories.

Fourth, required-item configuration limited item-level skipping despite the information page stating that questions could be skipped. Future surveys should align technical settings with the information sheet and implement branching logic more consistently.

Fifth, item 18 combined “fill” and “replace,” preventing separate estimates of supplementation and substitution. A revised instrument should ask these as distinct questions and should distinguish routine feedback, encouragement, relational care, and support for serious distress.

Sixth, the instrument grouped heterogeneous AI systems and language-learning tasks. Tool categories captured broad patterns of use but not model version, prompt design, feedback accuracy, or the instructional conditions surrounding each interaction. Multiple-response items also indicated whether an option was selected, not its priority, intensity, or causal importance. Future studies should combine tool-specific logs or task sampling with validated affect measures and repeated observations so that the timing and quality of a putative scaffold can be related to later engagement and performance.

Finally, open-ended responses were not analyzed because a formal qualitative protocol, independent coding procedure, and reliability assessment were not established before reporting. Future mixed-methods research should preregister sampling and coding procedures, retain an auditable consent process, and integrate qualitative evidence only after independent coding and transparent adjudication.

The operational model should therefore be treated as a framework for measurement development rather than a validated scale. A next step would combine cognitive interviews and expert content review with exploratory and confirmatory analyses in independent samples, followed by tests of measurement invariance across educational stages and AI-use experience. Such work should preserve the conceptual separation among affordances, attributed functions, affective outcomes, and teacher support; collapsing them into a single score would obscure the boundary the present study was designed to examine.

## Conclusion

In this adult convenience sample, helplessness, fatigue, and anxiety were common reported challenges in L2 English learning. Many respondents attributed affective support to AI, especially through immediate feedback and perceived relief of pressure or anxiety. Perceived AI support was moderately associated with reported enhancement of motivation and interest, but the cross-sectional design does not establish causation.

The findings support a bounded, perception-based account of AI-mediated affective support, which can be interpreted cautiously through affective scaffolding theory: AI can be experienced as a technological component of a supportive learning environment without possessing genuine emotion or replacing relational teaching. The most defensible pedagogical direction is complementarity—AI for accessible, repeatable, low-stakes support and teachers for contextual interpretation, human relationships, and accountable judgment. Stronger conclusions require representative samples, validated measures, properly documented consent, improved branching and item wording, and longitudinal or experimental designs.

### Generative AI use disclosure

OpenAI ChatGPT/Codex was used during revision to assist with English-language drafting, document formatting, and computational reproduction of aggregate frequency and chi-square calculations. The author checked the source data, statistical outputs, references, and final text and accepts full responsibility for the submitted work.

## Supporting information

S1 Data Aggregate frequencies, contingency tables, expected counts, and reproducible chi-square calculations for the adult analytical sample (N = 420). (XLSX)

S1 File English translation and analytic map of the Chinese-language questionnaire. (DOCX)
