# Supplementary material for: Learner perceptions of AI-mediated affective support in L2 English learning: A cross-sectional survey
Source: PLoS One. 2026 Oct 27;21(10):e0360925. doi: 10.1371/journal.pone.0360925 (PMC13712676; doi:10.1371/journal.pone.0360925)
Supplement: Supplementary file 1 [file pone.0360925.s002.docx]

**S1 File. English Translation and Analytic Map of the Chinese-Language Questionnaire**

The survey was administered in Chinese. This English translation is provided for transparency. It preserves the substantive wording and response options used for analysis. The public copy omits the researcher’s telephone number. The original survey did not include a separate consent checkbox; adults indicated electronic implied consent by proceeding to submit after reading the information page.

# Translated information page

Dear participant: This survey seeks to understand new developments and needs in foreign-language learning among Chinese university learners, with a focus on affective support perceived when using AI tools such as ChatGPT.

Participation was described as voluntary. The information page stated that participants could withdraw and that responses would be used only for statistical analysis and academic research and reported in aggregate. It stated that names and student numbers would not be collected. Researcher contact: Teacher Zhuo, School of Foreign Studies, Fuzhou University; email: joyzhuo18@126.com.

Implementation note for reproducibility: required closed-ended fields did not technically allow item-level skipping, although participants could leave before submission. The survey platform also recorded technical metadata automatically; these fields were removed before analysis.

# Items and response options

**Sex** [Single choice]

• Male

• Female

**Age group** [Single choice]

• Under 18

• 18–25

• 26–30

• 31–40

• 41–50

• 51–60

• Over 60

**Q1. Institution** [Free text]

**Q2. Educational stage** [Single choice]

• Undergraduate year 1

• Undergraduate year 2

• Undergraduate year 3

• Undergraduate year 4

• Master’s year 1

• Master’s year 2

• Master’s year 3

• Doctoral year 1

• Doctoral year 2

• Doctoral year 3

• Doctoral year 4 or above

• Postdoctoral

**Q3. Major/field** [Free text]

**Q4. English level (select the highest credential currently held)** [Single choice]

• Gaokao English >125

• CET-4

• CET-6

• TEM-4

• TEM-8

**Q5. What are your most salient emotional challenges in foreign-language learning?** [Multiple response]

• Anxiety

• Loneliness

• Fatigue

• Helplessness

• Other

**Q6. In which contexts do you usually experience these negative emotions?** [Multiple response]

• Language classroom

• After-class homework

• Practice activities

**Q7. Explain the main reasons for negative emotions in foreign-language learning and whether you think AI can help reduce them.** [Free text]

**Q8. Do you think AI (e.g., ChatGPT) can provide affective support and alleviate negative emotions in foreign-language learning?** [Single choice]

• Yes

• No

**Q9. When facing learning challenges, have you used AI to help complete tasks and reduce negative emotions?** [Single choice]

• Yes, sometimes/often

• No, never

**Q10. Which AI-assisted learning tools do you mainly use? (Skip if none.)** [Multiple response]

• Translation software (e.g., Google Translate, DeepL)

• Speech-recognition software (e.g., Siri, NaturalReader, Cortana)

• Language-learning apps (e.g., Duolingo, Ucampus AI, HelloTalk)

• International chatbot (ChatGPT)

• Domestic chatbots (e.g., ERNIE Bot, Doubao, DeepSeek)

• Other

**Q11. Through which mechanisms do you think AI mainly provides affective support?** [Multiple response]

• Immediate feedback

• Personalized learning plans

• Visualization of learning progress

• Encouragement and reward mechanisms

• Other

**Q12. What affective support does AI provide for your English learning?** [Multiple response]

• Reduces pressure and anxiety

• Enhances learning interest

• Fills gaps in teacher affective support and encouragement

• Simulates peer support

• Enhances confidence in foreign-language learning

• Other

**Q13. Through affective support, what effect does AI have on your foreign-language learning motivation?** [Single choice]

• Enhances motivation

• Reduces motivation

• No effect

**Q14. Through affective support, what effect does AI have on your foreign-language learning interest?** [Single choice]

• Enhances interest

• Reduces interest

• No effect

**Q15. How often do you currently use AI to assist English learning?** [Single choice]

• Several times daily

• Once daily

• 3–5 times weekly

• 1–2 times weekly

• Rarely

**Q16. How long is each AI-use session?** [Single choice]

• Less than 30 minutes

• 30–60 minutes

• More than 1 hour

**Q17. How satisfied are you overall with the AI-assisted learning tools you currently use?** [Single choice]

• Very satisfied

• Satisfied

• Neutral

• Dissatisfied

• Very dissatisfied

**Q18. Do you think AI might fill or replace the affective support provided by foreign-language teachers?** [Single choice plus explanation if No]

• Yes

• No; please explain

**Q19. With AI assistance, do you still need and expect teachers to increase their affective support?** [Single choice plus explanation if Yes]

• Yes; please specify the type of support

• No

**Q20. What are your expectations for the future affective-scaffolding functions of AI in foreign-language learning?** [Free text]

# Analytic notes

• The formal article analyzes closed-ended and multiple-response items only.

• Q7, the explanation fields attached to Q18 and Q19, and Q20 are not used for formal qualitative claims.

• Q10 and Q15–Q17 use n = 356 self-reported AI users.

• Q11–Q14 use N = 420 because the platform required later answers even from non-users.

• Q18 combines “fill” and “replace”; affirmative responses cannot be interpreted as support for complete teacher replacement.

• The instrument is researcher-developed and categorical. It is not a validated psychometric scale.
